# Are we validly assessing major depression disorder risk and associated factors among mothers of young children? A cross-sectional study involving home visitation programs

**DOI:** 10.1371/journal.pone.0209735

**Published:** 2019-01-07

**Authors:** Arthur H. Owora, Hélène Carabin, Tabitha Garwe, Michael P. Anderson

**Affiliations:** 1 Department of Public Health, Falk College, Syracuse University, Syracuse, New York, United States of America; 2 Department of Biostatistics and Epidemiology, University of Oklahoma Health Sciences Center, Oklahoma City, Oklahoma, United States of America; University of Toronto, CANADA

## Abstract

Failure to account for misclassification error accruing from imperfect case-finding instruments can produce biased estimates of suspected major depression disorder (MDD) risk factor associations. The objective of this study was to estimate the impact of misclassification error on the magnitude of measures of association between suspected risk factors and MDD assessed using the Center of Epidemiological Studies on Depression—Short Form during the prenatal and postnatal periods. Baseline data were collected from 520 mothers participating in two home visitation studies in Oklahoma City between 2010 and 2014. A Bayesian binomial latent class model was used to compare the prevalence proportion ratio (PPR) between suspected risk factors and MDD with and without adjustment for misclassification error and confounding by period of MDD symptom on-set. Adjustment for misclassification error and confounding by period of MDD on-set (prenatal vs postnatal) showed that the association between suspected risk factors and MDD is underestimated (-) and overestimated (+) differentially in different source populations of low-income mothers. The median bias in the magnitude of PPR estimates ranged between -.47 (95% Bayesian Credible Intervals [BCI]: -10.67, 1.90) for intimate partner violence to +.06 (95%BCI: -0.37, 0.47) for race/ethnicity among native-born US residents. Among recent Hispanic immigrants, bias ranged from -.77 (95%BCI: -15.31, 0.96) for history of childhood maltreatment to +.10 (95%BCI: -0.17, 0.39) for adequacy of family resources. Overall, the extent of bias on measures of association between maternal MDD and suspected risk factors is considerable without adjustment for misclassification error and is even higher for confounding by period of MDD assessment. Consideration of these biases in MDD prevention research is warranted.

## Introduction

Major depression disorder (MDD) is prevalent among women and is correlated with poor health and psychosocial outcomes for both mothers and the children under their care [1, 2]. Depressed women have poorer health, psychosocial outcomes and lower quality of life than non-depressed women [3, 4]. Children of depressed mothers are more likely to exhibit general difficulties in functioning, attachment problems, and develop mood disorders than children of non-depressed mothers [5, 6]. These negative outcomes for both mother and child make the accuracy of MDD detection critical for the identification of malleable risk factors with intervention potential that could aid MDD prevention efforts.

The results of five meta-analyses of factors associated with maternal MDD have classified the magnitude of effects as being small (obstetric and pregnancy complications, cognitive attributions, relationship problems and social economic status), moderate (childcare stress, low self-esteem, and neuroticism) and strong (stressful life events, lack of social support, maternal age and a history of depression)[7–11]. Most of these factors are prevalent among socially disadvantaged women, potentially resulting in large numbers of mothers developing MDD and consequently a substantial public health burden.

To address this issue, all 50 states have now adopted home visitation programs to support socially disadvantaged mothers and enhance efforts to improve maternal and child health outcomes (including reduction of the burden of MDD)[2, 12, 13]. In order to maximize the potential benefit of home visitation programs, it is essential to accurately identify which factors are associated with MDD, so that modifiable factors are intervened on to reduce the risk of MDD. Significant bivariate associations between MDD and trauma history[14, 15], young maternal age[12, 14], history of intimate partner violence[16], parent distress[16, 17], history of child abuse[14, 15] and anxiety/distrust problems[12] have been reported in studies conducted among high risk populations in home visitation settings. However, these results may be biased by the presence of confounders such as period of MDD assessment (prenatal versus postnatal). Moreover, most of these associations are based on MDD assessments using imperfect (i.e. less than 100% sensitivity and specificity) case-finding instruments.

MDD case-finding instruments rely on the self-report of MDD-related symptoms which is particularly prone to misclassification error because mothers are likely to report what is expected of them (social desirability bias), may interpret MDD symptom descriptions differently according to their cultures[18–20] and may confuse MDD symptoms with normal symptoms felt by women during the prenatal and postnatal period [21]. Additionally, the reliability of MDD case-finding instrument symptom assessments within and between reporters (mothers) vary widely [13, 22–24].

The validity (i.e. sensitivity and specificity) of MDD case-finding instruments also vary depending on cutoff-point thresholds and period of MDD assessment [25]. The resulting false positives and negatives (misclassification error) can over or under-estimate the prevalence of MDD [26] and consequently measures of association between risk factors and MDD [27, 28].

In the context of home visitation programs, MDD case-finding instruments (e.g. Center for Epidemiologic Studies Depression Scale–Short Form—*CESD-SF*) are conducted at home [29], which by itself could increase or reduce the level of misclassification error compared to structured diagnostic interviews (based on Diagnostic and Statistical Manual of Mental Disorders criteria for MDD) in a clinical setting. Indeed, it is possible that a mother would feel uncomfortable admitting to some of the symptoms measured in the MDD case-finding instrument in front of her children, partner or other household members. She may also feel less at ease admitting to such symptoms in front of a home visitor, who often lacks the training needed to identify and address depressive symptoms.

We recently applied Bayesian latent class methodology to estimate the impact of misclassification error on the estimated prevalence proportion of MDD among mothers of young children (0–5 years old) during prenatal and postnatal periods; our findings showed that combining these periods confounded bias estimates (i.e. amount by which the prevalence proportion was under- or over-estimated) [26]. To our knowledge, no study has examined the individual or joint impact of misclassification error and confounding by period of MDD assessment on measures of association between suspected risk factors and MDD in home visitation settings or among women in general. Therefore, the objective of our study is to extend our previous work [26] by examining (1) the impact of CESD-SF case-finding instrument misclassification error, and (2) the joint impact of misclassification error and confounding by period of MDD assessment on measures of association between MDD and suspected risk factors among mothers of young children enrolled at two home visitation program study sites. We also compare and contrast the impact of these issues between the two study sites.

## Methods and procedures

Oklahoma University Health Sciences Center IRB approved study related procedures and activities.

### Data source

This study is a secondary analysis of the baseline component of two ongoing home visitation studies targeting mothers of young children in Oklahoma, which targeted two distinct populations. One study site, SITE I, targeted a diverse racial/ethnic mix of low-income mothers while the other, SITE II, targeted a population of low-income, first and second-generation Hispanic immigrant mothers. The objective of these studies was to examine the effectiveness of evidence-based home visitation programs on improving parent-infant/child outcomes such as prevention of child maltreatment, reducing parent substance and alcohol abuse, intimate partner violence and parenting stress. The two study populations had very different distributions of risk factors and frequency of MDD [26], which resulted in important differences in the measures of association between the sites. This led to the decision to report the results separately for the two study sites.

### Study design

The baseline components of two conveniently chosen ongoing home visitation study sites were analyzed as cross-sectional designs. Baseline data were collected between December 2010 and December 2014 from consecutively enrolled mothers of children aged five years or younger.

### Study setting

Participants in the ongoing home visitation studies were recruited from physician offices, medical centers, faith-based organizations, schools, mental health agencies, other HV programs, and welfare programs (e.g. Women, Infants and Children program) in Oklahoma City. At baseline, prior to enrollment in home visitation services, mothers completed a battery of questionnaires including questions on basic socio-demographic information, family and social supports, history of intimate partner violence, childhood maltreatment and MDD symptoms (using the CESD-SF). These questionnaires were completed using an audio computer assisted self-interview (ACASI) application with assistance from a trained data collector if needed.

### Study participants

The three inclusion criteria for the current analysis were: 1) being a mother, 2) having at least one child who was five years old or younger at baseline (pregnant women with no other children were ineligible), and 3) having a completed *CESD-SF* at baseline. For our study analyses, because we focused on baseline data, miscarriages, stillbirth or death of children after baseline assessments did not influence our study findings.

Informed verbal and written consent was obtained from all eligible study participants. For minors, involved in the study, we obtained their verbal and written consent as well as that of their parents or guardians. Differences in the distribution of study characteristics at the two study sites (SITE I and II) were examined and are summarized elsewhere [26].

### MDD outcome ascertainment

The operational definition of MDD status was determined using the CESD-SF. Here, the results of the CESD-SF are not used as a diagnosis of MDD but rather are indicative of the presence of MDD-related symptoms. Only a qualified health professional can diagnose MDD through a more thorough diagnostic interview. Mothers at baseline completed the CESD-SF in English or Spanish prior to initiation of home visitation services. The CESD-SF is a 12-item multi-choice instrument designed to assess if an individual has exhibited some level of MDD-related symptoms during the past week with scores ranging from 0 to 36 [29]. The CESD-SF is derived from the 20-item scale originally developed as a measure of MDD-related symptoms in community-based epidemiological studies [29, 30]. This instrument has been used to screen for MDD during the prenatal, postnatal and combined periods [31–33]. Having moderate MDD symptoms was defined operationally as a CESD-SF score of 10 or above [29]. This threshold is comparable to the cut-off point threshold of 16 (≥16) on the CESD20 [29]. The CESD-SF showed good internal consistency (Cronbach’s alpha) for the assessment of depression-related symptoms at both SITE I (0.92) and II (0.93).

We operationally define periods of MDD assessment among study participants as ‘prenatal’ or ‘postnatal’ depending on whether the CESD-SF baseline assessment were administered during pregnancy or after childbirth, respectively. Eligible pregnant women or mothers had at least one child who was five years old or younger. In this study, the term the ‘misclassification error’ refers to the incorrect classification of an individual as either a false positive or false negative by the CESD-SF. ‘Confounding by period of MDD assessment’ refers to the distortion of the relationship (measured by the prevalence proportion ratio) between an individual’s true MDD status and suspected risk factor as a function of the differential proportion distribution of assessments (prenatal versus postnatal) among individuals with and without a risk factor of interest.

### Measurement and definition of suspected risk factors

Information on suspected MDD risk factors (e.g. socio-demographic characteristics, adequacy of family resources and social support, history of childhood maltreatment and intimate partner violence) is derived from self-report questionnaires administered at baseline. Factors that have been previously identified as risk factors for MDD among mothers were examined [2]. Self-reported race and ethnicity was categorized as White, Black, and Other (i.e. Hispanic, Native American and Asians). The adequacy of resources in households with children and the degree to which a respondent’s social relationships provide various dimensions of social support were measured using the 30-items Family Resources Scale-Revised [34] and the 12-items Social Provisions Scale-Short Form [35], respectively. A history of childhood maltreatment was determined based on a self-report of any child abuse or neglect event that occurred during a mother’s childhood. Presence of intimate partner violence (among mothers who reported being in a relationship at baseline) was determined based on a self-report of physical assault by a partner (i.e. physical assault victimization sub-scale) in the past six months on the Conflict Tactics Scale Second version [36].

### Statistical analysis

Measures of association (estimated by prevalence proportion ratios) were generated for each study site separately since they targeted very different populations of mothers and the impact of the different risk factors were expected to vary between sites. Prevalence proportion ratios (PPRs) were obtained by comparing prevalence proportions between sub-groups defined by levels or categories of a characteristic/risk factor to a reference sub-group (i.e. PPR = prevalence proportion in sub-group 1/ prevalence proportion in sub-group 2 [reference group]).

The Bayesian latent class models used for the estimation of prevalence proportions are described in our previous study [26]. Briefly, the probability of a test result and probability of MDD are related via conditional probability concepts as follows: P (T^+^) = P (T^+^|D^+^) P (D^+^) + P (T^+^|D^-^) P (D^-^) where P(T^+^) corresponds to the proportion of participants testing positive for MDD (observed prevalence), P (T^+^|D^+^) to the sensitivity of the test, P (T^+^|D^-^) corresponds to one minus the specificity of the test, and P (D^+^) to the true prevalence of MDD.

Therefore, P (D+) = (P (T^+^|D^+^) + P (T^-^|D^+^))/ (P (T^+^|D^+^) + P (T^+^|D^-^) + P (T^-^|D^-^) + P (T^-^|D^+^)). In the absence of a gold standard, this equation cannot be solved directly since there are three unknown parameters (sensitivity, specificity and true prevalence) but only one degree of freedom from the observed data (number of positive tests out of the total sample size fixes the number testing negative). A Gibbs sampler is used to randomly sample the joint posterior density of the parameters of interest (sensitivity, specificity and true prevalence) which is the product of the likelihood of the data (latent and observed) and prior distributions of all parameters. The samples from the joint posterior distribution are then used to generate the marginal posterior densities of prevalence proportion, sensitivity and specificity parameters.

For PPR estimation, all investigated risk factors were dichotomized. The FRS and SPS scales were dichotomized based on the median value of the participants’ scores to represent high versus low family resources and social support respectively. Marital status was dichotomized into married or living with a partner versus single (i.e. never married, divorced, separated or widowed). Being born in the US, having healthcare insurance, history of childhood maltreatment and history of intimate partner violence were dichotomized as yes/no variables. Educational level was dichotomized as attainment of high school or General Education Development (GED) equivalent or higher versus less than a high school diploma/GED. Employment status was dichotomized as full or part-time versus unemployed and maternal age was dichotomized (based on the median age of 25 years old).

For each sub-group defined by the presence or absence of a characteristic, the MDD prevalence proportions were obtained using a Bayesian latent class analysis model in the absence of a ‘gold standard’ with and without adjustment for CESD-SF misclassification error and confounding by period of MDD assessment. The median estimate of the posterior density distribution of each PPR for each investigated characteristic including its 95% Bayesian Credible Interval (95%BCI) were obtained separately. The 95% BCIs give us the 95% probability coverage range of median PPR values. S1 File shows the WinBUGS program code used to generate these estimates.

Convergence was assessed by examining the plots of chain histories, the results from the Geweke test [37], Heidelberger-Welch stationary test [38], and Gelman-Rubin test [39]. WinBUGS software (version 1.4.3, MRC Biostatistics Unit, Cambridge, UK) was used to implement the Bayesian latent class models [40].

### Handling of missing data

WinBUGS automatically simulates values for missing observations according to the specified likelihood distribution (in our case—binomial distribution), conditional on the current values of all relevant unknown parameters (i.e. prevalence proportions and PPRs) described above.

### Prior distributions

Informative prior estimates of CESD-SF sensitivity and specificity among mothers of young children were based on a previous systematic review where the posterior estimates of sensitivity and specificity were obtained for CESD20 with consideration for misclassification error in the reference standards [41]. Only one study, Tandon et al [33], provided prenatal and postnatal period specific diagnostic performance estimates. The period-specific posterior estimates derived from a Bayesian Hierarchical Summary Receiver-Operating Characteristic Curve (HSROC) meta-analysis model [41] are considered as prior information (Table 1). Informative priors are a key part of Bayesian inference that represent information about an uncertain parameter (in our case the sensitivity and specificity estimates of the CESD-SF). These priors are combined with the probability distribution of new data (True Positives, True Negatives, False Positives, and False Negatives) to yield a posterior distribution of case-finding instrument sensitivity and specificity estimates from which summary estimates of parameters (i.e. median prevalence proportion, PPRs and 95% Bayesian Credible Intervals [BCI]) are estimated.

**Table 1:** Mean and standard deviation of prior distributions used for CESD-SF parameters (with and without correcting for reference standard misclassification error) used in Bayesian latent class models.

Study: Assessment Period	Estimates	Mean (SD) priors ignoring misclassification in the reference standard^a^	Mean (SD) priors adjusted for misclassification error in the reference standard^b^
Prenatal	Sensitivity	0.73(0.12)	0.89(0.11)
	Specificity	0.83(0.07)	0.91(0.07)
Postnatal	Sensitivity	0.82(0.07)	0.93(0.07)
	Specificity	0.85(0.05)	0.93(0.05)
Combined	Sensitivity	0.67 (0.08)	0.77(0.12)
	Specificity	0.89(0.03)	0.97(0.03)

^a^Prior estimates from: Tandon SD, Cluxton-Keller F, Leis J, Le HN, Perry DF. A comparison of three screening tools to identify perinatal depression among low-income African American women. J Affect Disord. 2012; 136(1–2):155–62. doi: 10.1016/j.jad.2011.07.014.

^b^Prior estimates from: Owora AH, Carabin H, Reese J, Garwe T. Summary diagnostic validity of commonly used maternal major depression disorder case finding instruments in the United States: A meta-analysis. J Affect Disord. 2016; 205:335–43. doi: 10.1016/j.jad.2016.08.014.

### Bias estimation

Bias was measured as the median and 95% Bayesian Credible Interval (95%BCI) of the difference between the observed PPR [P (T^+^)_1_/ P (T^+^)_2_] and the misclassification error-adjusted PPR [P (D^+^)_1_/ P (D^+^)_2_]. The 95%BCI is interpreted as the 95% probability coverage of the true median bias [Bias = P (T^+^)_1_/ P (T^+^)_2_ minus P (D^+^)_1_/ P (D^+^)_2_] value assuming adequate statistical model and prior distribution assumptions. Two estimates of misclassification error-adjusted PPR estimates were examined.

The first misclassification error-adjusted PPR estimate (without adjustment for confounding by period of MDD assessment) was generated by (a) combining data across prenatal and postnatal periods, and (b) using prior values based on combined periods to compare MDD prevalence proportions in risk factor sub-groups (i.e. prevalence proportion_1_ / prevalence proportion_2_)

The second misclassification error-adjusted PPR estimate (with adjustment for confounding by period of MDD assessment) was generated by (a) separating data into prenatal versus postnatal period MDD assessments and (b) using period-specific prior values to compare MDD prevalence proportions in risk factor sub-groups (i.e. prevalence proportion_1(prenatal + postnatal)_ / prevalence proportion_2(prenatal + postnatal)_).

## Results

### Study population

As of December 31 2014 (date analytical sample was obtained), 429 and 687 referrals had been received by SITE I and II, respectively; 318 (74%) and 467 (68%) were eligible. Among eligible participants, 179 (56%) and 341 (73%) mothers were successfully recruited into the study and completed baseline assessments at SITE I (low income mothers who were US residents) and SITE II (recent Hispanic immigrant mothers), respectively. Most socio-demographic characteristics differed between the two study sites [26]. Specifically, there were older (>25 years old), more married (or living with a partner), less high school graduates, more foreign-born mothers at SITE II than SITE I (*p*<0.05).

### Observed measures of association (PPR) between maternal MDD and suspected risk factors

The measures of association (i.e. PPR) of investigated risk factors were quite different between the study sites (Tables 2 and 3: crude estimates). Being single, having a history of intimate partner violence, low levels of social supports and family resource were associated with a higher prevalence of MDD at SITE I (Table 2). At SITE II, in addition to risk factors identified at SITE I, having a history of childhood maltreatment and unemployment were also associated with MDD (Table 3). Overall, the prevalence of MDD was 50% higher at SITE I than II (PPR: 1.50; 95% BCI: 1.23; 1.83).

**Table 2:** Crude and misclassification error adjusted measures of association between suspected risk factors and MDD based on CESD-SF self-reports across socio-demographic factors at SITE I.

	SITE I [N = 179] Prevalence Proportion Ratio [PPR]			BIAS 95% BCI	
Characteristic	Crude^a^ [PPR; 95% BCI]	ME adjusted^b^ [PPR; 95% BCI]	ME adjusted^c^ [PPR; 95% BCI]	Crude^a^ - ME adjusted^b^	Crude^a^ - ME adjusted^c^
Period					
Prenatal	Ref	Ref	Ref		
Postnatal	1.04[0.75, 1.64]	0.93 [0.64, 1.50]	0.97 [0.44, 1.73]	-0.02 [-0.64, 0.55]	-0.02 [-0.82, 0.65]
Maternal Age					
<25 years	Ref	Ref	Ref		
≥25 years	1.05 [0.79, 1.37]	1.04 [0.79, 1.40]	1.07 [0.77, 1.59]	-0.01 [-0.42, 0.44]	-0.02 [-0.59, 0.45]
Race/Ethnicity					
NH, White	Ref	Ref	Ref		
NH, Black	0.54[0.33, 0.88]	0.54 [0.28, 0.85]	0.51 [0.22, 0.87]	0.02 [-0.38, 0.42]	0.06 [-0.37, 0.47]
Others	0.81[0.61,1.06]	0.81 [0.61, 1.08]	0.80 [0.57, 1.21]	-0.01 [-0.33, 0.33]	0.02 [-0.35, 0.37]
Marital Status					
Married/Living with a Partner	Ref	Ref	Ref		
Single	1.56 [1.13, 2.15]	1.58 [1.15, 2.38]	1.73 [1.19, 3.28]	-0.04[-0.93, 0.71]	-0.16 [-1.75, 0.70]
IPV					
No	Ref	Ref	Ref		
Yes	2.10 [1.72, 2.56]	2.17 [1.21, 4.80]	2.60 [1.27, 12.87]	-0.08 [-2.78, 1.90]	-0.47 [-10.67, 1.90]
History of CM					
No	Ref	Ref	Ref		
Yes	1.26 [0.95, 1.70]	1.26 [0.94, 1.76]	1.34 [0.95, 2.16]	-0.01 [-0.60, 0.53]	-0.07 [-0.93, 0.55]
HS/GED					
No	Ref	Ref	Ref		
Yes	1.33 [1.16, 1.51]	1.34 [0.95, 2.18]	1.48 [0.96, 3.11]	-0.03 [-0.91, 0.76]	-0.10 [-1.76, 0.84]
Employment					
Unemployed	Ref	Ref	Ref		
Full-time/Part-time employed	0.77 [0.66, 0.87]	0.77 [0.51, 1.08]	0.77 [0.48, 1.12]	0.01 [-0.39, 0.40]	0.03 [-0.40, 0.44]
HealthCare Insurance					
No	Ref	Ref	Ref		
Yes	1.47[1.03, 2.20]	1.49 [1.03, 2.50]	1.30 [0.82, 3.24]	-0.03 [-1.12, 0.86]	-0.07 [-2.00, 0.83]
Place of birth					
Foreign-born	Ref	Ref			
US born	1.67 [1.03, 3.31]	1.71 [1.04, 3.86]	2.00 [1.09, 8.38]	-0.06 [-2.18, 1.54]	-0.25 [-6.57, 1.78]
Social Support					
1^st^ & 2^nd^ Quartile	Ref	Ref	Ref		
3^rd^ & 4^th^ Quartile	0.65[0.49,0.85]	0.64 [0.47, 0.85]	0.60 [0.41, 0.83]	0.01 [-0.25, 0.27]	0.05 [-0.23, 0.32]
Family Resource					
1^st^ & 2^nd^ Quartile	Ref	Ref	Ref		
3^rd^ & 4^th^ Quartile	0.76 [0.59, 0.99]	0.76 [0.56, 1.01]	0.75 [0.53, 1.07]	<0.01 [-0.31, 0.31]	0.03 [-0.34, 0.36]

ME, misclassification error; PPR, Prevalence proportion ratio; CM, Childhood maltreatment; HS, High School; GED, General Equivalency Diploma; NH, Non-Hispanic; IPV, Intimate partner violence

^a^crude PPR estimates are based on non-informative priors and ignore both misclassification error (i.e. assume 100% CESD-SF sensitivity and specificity) and confounding by period of MDD assessment

^b^misclassification error adjusted PPR estimates ignoring confounding by period of MDD assessment

^c^misclassification error adjusted PPR estimates adjusted for confounding by period of MDD assessment

**Table 3:** Crude and misclassification error adjusted measures of association between suspected risk factors and MDD based on CESD-SF self-reports across socio-demographic factors at SITE II.

	SITE II [N = 341] Prevalence Proportion Ratio [PPR]			BIAS 95% BCI	
Characteristic	Crude^a^ [PPR; 95% BCI]	ME adjusted^b^ [PPR; 95% BCI]	ME adjusted^c^ [PPR; 95% BCI]	Crude^a^ - ME adjusted^b^	Crude^a^ - ME adjusted^c^
Period					
Prenatal	Ref	Ref	Ref		
Postnatal	1.07[0.95, 1.23]	1.09 [0.72, 1.84]	0.91 [0.21, 2.07]	-0.01 [-0.81, 0.68]	-0.02 [-1.19, 0.81]
Maternal Age					
<25 years	Ref	Ref	Ref		
≥25 years	0.76[0.57, 1.02]	0.75 [0.53, 1.03]	0.72 [0.45, 1.15]	0.02 [-0.32, 0.35]	0.04 [-0.42, 0.44]
Marital Status					
Married/Living with a Partner	Ref	Ref	Ref		
Single*	1.61[1.16, 2.16]	1.67 [1.67, 2.38]	1.84 [1.26, 3.88]	-0.06 [-0.86, 0.63]	-0.24 [-2.29, 0.56]
IPV					
No	Ref	Ref	Ref		
Yes	1.75[1.56, 1.97]	1.85 [1.26, 2.91]	2.09 [1.32, 5.98]	-0.09 [-1.25, 0.82]	-0.35 [-4.23, 0.73]
History of CM					
No	Ref	Ref	Ref		
Yes	2.19 [1.94, 2.47]	2.33 [1.64, 4.52]	3.15 [1.89, 17.74]	—0.19 [-2.38, 0.98]	-0.77 [-15.31, 0.96]
HS/GED					
No	Ref	Ref	Ref		
Yes	0.97 [0.71, 1.34]	0.97 [0.65, 1.37]	1.02 [0.62, 2.05]	-0.01[-0.47, 0.47]	-0.02 [-1.07, 0.52]
Employment					
Unemployed	Ref	Ref	Ref		
Full-time/Part-time employed	1.56[1.13,2.07]	1.61 [1.16, 2.29]	1.77 [1.20, 3.63]	-0.05 [-0.82, 0.63]	-0.21 [-2.07, 0.55]
HealthCare Insurance					
No	Ref	Ref	Ref		
Yes	0.88[0.64, 1.24]	0.89 [0.58, 1.28]	1.03 [0.60, 1.65]	0.01 [-0.46, 0.48]	-0.01 [-0.71, 0.57]
Place of birth					
Foreign-born	Ref	Ref	Ref		
US born	1.51 [0.93, 2.14]	1.52 [0.91, 2.26]	1.66 [0.95, 3.17]	—0.02 [-0.95, 0.85]	-0.17 [-1.73, 0.78]
Social Support					
1^st^ & 2^nd^ Quartile	Ref	Ref	Ref		
3^rd^ & 4^th^ Quartile	0.70 [0.50, 0.97]	0.65 [0.43, 0.89]	0.64 [0.33, 1.22]	0.08 [<0.01, 0.15]	0.06 [-0.15, 0.26]
Family Resource					
1^st^ & 2^nd^ Quartile	Ref	Ref	Ref		
3^rd^ & 4^th^ Quartile	0.44[0.30,0.62]	0.40 [0.20, 0.60]	0.35 [0.11, 0.58]	0.04 [-0.19, 0.32]	0.10 [-0.17, 0.39]

ME, misclassification error; PPR, Prevalence proportion ratio; CM, Childhood maltreatment; HS, High School; GED, General Equivalency Diploma; NH, Non-Hispanic; IPV, Intimate partner violence

^a^crude PPR estimates are based on non-informative priors and ignore both misclassification error (i.e. assume 100% CESD-SF sensitivity and specificity) and confounding by period of MDD assessment

^b^misclassification error adjusted PPR estimates ignoring confounding by period of MDD assessment

^c^misclassification error adjusted PPR estimates adjusted for confounding by period of MDD assessment

### Magnitude of bias in measures of association (PPR) ignoring confounding by period of MDD assessment

**Fig 1:**
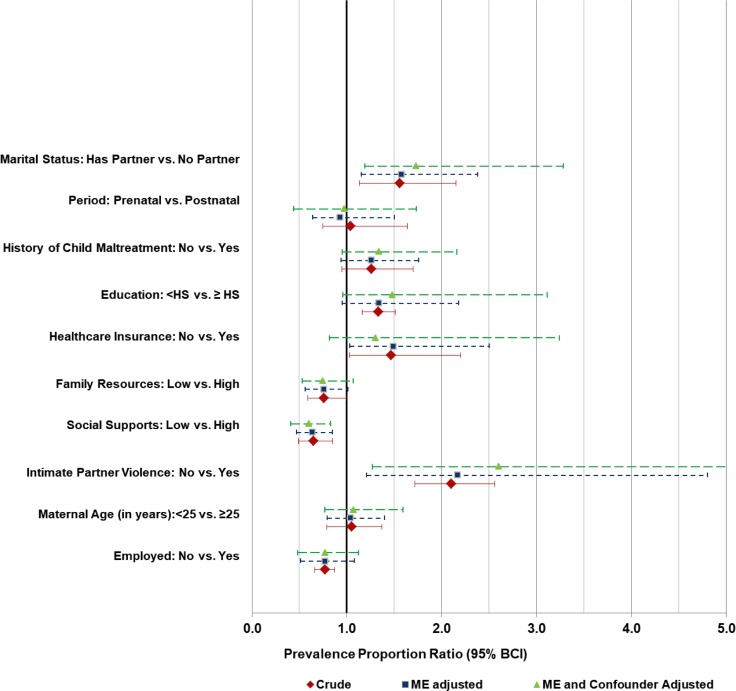
Crude and adjusted measures of association between suspected risk factors and MDD assessed by the CESD-SF at SITE I. Red diamond symbol—crude PPR estimates are based on non-informative priors and ignore both misclassification error (i.e. assume 100% CESD-SF sensitivity and specificity) and confounding by period of MDD assessment. Dark blue square symbol—misclassification error adjusted PPR estimates ignoring confounding by period of MDD assessment. Light green triangle—misclassification error adjusted PPR estimates adjusted for confounding by period of MDD assessment. HS- High school (or GED equivalent). ME- misclassification error.

**Fig 2:**
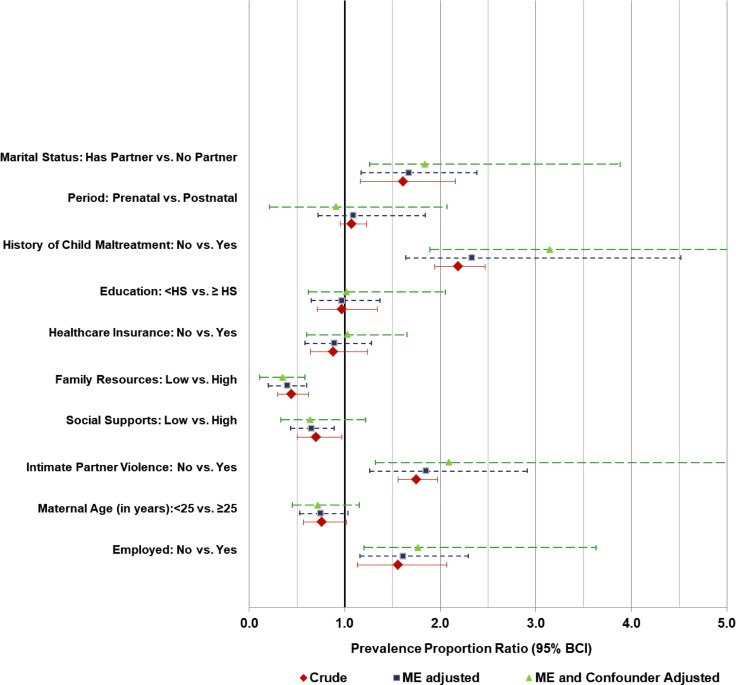
Crude and adjusted measures of association between suspected risk factors and MDD assessed by the CESD-SF at SITE II. Red diamond symbol—crude PPR estimates are based on non-informative priors and ignore both misclassification error (i.e. assume 100% CESD-SF sensitivity and specificity) and confounding by period of MDD assessment. Dark blue square symbol—misclassification error adjusted PPR estimates ignoring confounding by period of MDD assessment. Light green triangle—misclassification error adjusted PPR estimates adjusted for confounding by period of MDD assessment. HS- High school (or GED equivalent). ME- misclassification error.

When all data were combined (i.e. prenatal and postnatal MDD assessments) and analyzed using diagnostic performance priors for the combined periods as a whole, the association between MDD and suspected risk factors seemed to be biased towards the null at both study sites (Tables 2 and 3). Figs 1 and 2 are a visual illustration of the median and 95%BCIs of risk factor associations examined at SITE I and II, respectively.

The prevalence of MDD was higher at SITE I than SITE II (PPR: 1.54; 95%BCI: 1.22, 2.00) indicating negligible bias (-0.04; 95% BCI: -0.56, 0.40).

For SITE I, the magnitude of bias was small but with substantial uncertainty for all the risk factors. The level of bias in risk factor measures of association (PPR) ranged from -0.08 (95%BCI: -2.78, 1.90) for intimate partner violence [under-estimation of PPR] to 0.02 (95%BCI: -0.38, 0.42) for race/ethnicity comparisons [over-estimation of PPR]. For level of education, employment status, and FRS, the adjustment for misclassification error shifted the posterior distribution of PPRs towards one and reduced their 95% probability coverage (95% BCI). Having a history of intimate partner violence (adjusted PPR: 2.17; 95%BCI: 1.21; 4.80), healthcare insurance (adjusted PPR: 1.49; 95%BCI: 1.03; 2.50), being born in the US (adjusted PPR: 1.71; 95%BCI: 1.04; 3.86) and being a single mother (adjusted PPR: 1.58; 95%BCI: 1.15; 2.38) were associated with a higher prevalence of MDD. High scores of social support (SPS) were associated with a lower prevalence of MDD (adjusted PPR: 0.65; 95%BCI: 0.47; 0.85).

For SITE II, the magnitude of bias estimates were generally higher ranging from 0.08 (95%BCI: <0.01, 0.15) for social support to -0.01 (95%BCI: -0.81; 0.68) for period of MDD assessment. Although, adjustment for misclassification error increased uncertainty (wider 95% BCI), all crude associations (i.e. crude PPRs) remained after misclassification error adjustment. For example, the association between MDD and employment status was 1.56 (95%BCI: 1.13; 2.07) for the crude PPR and 1.61 (95%BCI: 1.16; 2.29) for the adjusted PPR. Similar effects were observed for social support with crude PPR of 0.70 (95%BCI: 0.50; 0.97) and adjusted PPR of 0.65 (95%BCI: 0.42; 0.89). Other factors associated with a higher prevalence of MDD included: being single, a history of intimate partner violence, and childhood maltreatment. High scores of family resources (FRS) and social support (SPS) were associated with a lower prevalence of MDD (Table 3).

### Magnitude of bias in measures of association (PPR) adjusted for CESD-SF misclassification error and confounding by period of MDD assessment

Across the risk factors examined at both study sites, adjusting for the differential diagnostic performance of the CESD-SF during the prenatal and postnatal periods and the different proportions of mothers who had assessments in these periods showed that a higher magnitude of bias (Tables 2 and 3) was present than was observed when the analysis was conducted on the combined periods. The prevalence of MDD was higher at SITE I than SITE II after adjusting for both misclassification error and the confounding by period of MDD assessment (PPR: 1.66; 95%BCI: 1.28; 2.78) than when only misclassification error was adjusted for (adjusted PPR: 1.54; 95%BCI: 1.23, 2.00).

At SITE I, the bias in the PPR ranged from -0.47 (95%BCI: -10.67, 1.90) for intimate partner violence to -0.02 (95%BCI: -0.82, 0.65) for period of MDD assessment (Table 2). The high magnitude of bias for intimate partner violence resulted in an increase in the median PPR of nearly 23%, but also greater uncertainty (i.e. wider 95%BCI). Similar to the misclassification error-adjusted PPRs, PPRs adjusted for both confounding and misclassification error suggested a higher MDD prevalence among single mothers, those who reported intimate partner violence and White mothers (compared to Black mothers) with the exception of having healthcare insurance (Table 2). Before adjustment for confounding, there was an association between MDD and healthcare insurance (misclassification error-adjusted PPR: 1.49; 95%BCI: 1.03; 2.50; Bias: -0.03; 95%BCI: -1.12; 0.86); however, joint adjustment for confounding and misclassification error resulted in a 95% PPR estimate probability coverage that overlaps one (adjusted PPR: 1.30; 95%BCI: 0.82; 3.24; Bias: -0.07; 95%BCI: -2.00; 0.83).

At SITE II, the bias in the PPR estimates was generally larger and ranged from -0.77 (95%BCI: -15.31; 0.96) for history of childhood maltreatment to 0.10 (95%BCI: -0.17; 0.39) for adequacy of family resources. Similar to SITE I, bias in risk factor measures of association was high and most variable for intimate partner violence (-0.77; 95%BCI: -15.31; 0.96). Compared to misclassification error-adjusted median PPR estimates, joint adjustment for both misclassification error and confounding suggested a much higher prevalence of MDD among mothers that were single, had a history of intimate partner violence, low family resource scores, childhood maltreatment, and unemployed (Table 3). There was also more uncertainty in the joint misclassification error and confounding adjusted PPRs estimates (i.e. wider 95% BCIs). For some factors (e.g. social support), this increased uncertainty resulted in a 95% PPR estimate probability coverage whose posterior distribution overlapped one (misclassification error-adjusted PPR: 0.65; 95%BCI: 0.43; 0.89; Bias: 0.08; 95%BCI: <0.01; 0.15) versus misclassification error and confounding-adjusted PPR: 0.64; 95%BCI: 0.33, 1.22; Bias: 0.06; 95%BCI: -0.15; 0.26).

## Discussion

This study is the first to demonstrate that misclassification error and confounding by period of MDD assessment (prenatal versus postnatal) can introduce bias and considerable uncertainty in estimating the association between potential risk factors and MDD among two distinct groups of mothers involved in home visitation programs. This analysis extends our previous work (i.e. impact of misclassification error on MDD prevalence proportion estimates) [26] to encompass measures of association (PPR) comparing prevalence proportions.

Our previous work [26] showed that when using prenatal and postnatal period specific sensitivity and specificity of the CESD-SF, associated misclassification error led to nearly no bias in *prevalence* estimates. In contrast, ignoring differences in CESD-SF sensitivity and specificity between these periods showed considerable *MDD prevalence bias*. Our current analyses extend this previous work to show that misclassification error and confounding by period of MDD assessment can also lead to the underestimation in some (e.g. intimate partner violence at SITE I and II) and overestimation in other (e.g. period of MDD assessment at SITE I and II) *measures of risk-factor associations (i*.*e*. *Prevalence Proportion Ratios)* with varying magnitudes depending on the source population of interest.

Our current findings show that failure to account for the differential diagnostic performance of the CESD-SF during prenatal and postnatal periods and associated confounding effects can erroneously create an impression of negligible bias and uncertainty in estimation of measures of association than actually exists. Indeed, as evidenced by the generally larger median bias estimates for suspected MDD risk factor associations based on the joint-adjustment for misclassification error and confounding versus adjustment for misclassification error only, the strength of an association could be underestimated or overestimated. Our results also suggest that adjustment for misclassification error and confounding come with the cost of diminished precision of PPR estimates; however, this could be due to the small sample sizes in our stratified sub-group analysis as illustrated by the study site comparisons examining the same risk factors (where, SITE I has a smaller sample size [hence wider 95%BCI] than SITE II).

Differential diagnostic performance of the CESD-SF between prenatal and postnatal periods was reflected in the prior values, which were based on a study by Tandon et al [33]. The sensitivity and specificity values for the CESD20 were similar in the prenatal and postnatal period, but differed when these periods were combined. When data from these periods are combined, sensitivity is underestimated while the specificity is overestimated compared to the period-specific estimates. When period-specific adjustments are made for misclassification error and then combined, a lower proportion of false negatives is presumed. This results in a lower or negligible impact of misclassification error adjustments on the magnitude of MDD prevalence estimates in the risk factor sub-groups (i.e. risk present versus absent) across assessment periods.

In light of the misclassification error that could result from the use of case-finding instrument cut-point thresholds to identify, compare and contrast mothers with and without MDD-related symptoms, the use of continuous scores from these instruments should be explored as an alternative approach to investigate risk factor associations in future research. While measurement error (i.e. misclassification involving a continuous scale test result) cannot be ruled out even when using case-finding instrument scores, this approach can provide a useful dose-response gradient at different levels or categories of suspected risk factors. This is central to determining “safe”, “hazardous” or beneficial levels of modifiable factors with intervention potential.

The impact of confounding by period of MDD assessment on the extent of bias observed could be explained by the: 1) differential proportion distribution of mothers with a particular risk factor (characteristic) who had assessments in the prenatal versus postnatal periods and 2) differential diagnostic performance of the CESD-SF during these periods.

In our study, the impact of the differential proportion distribution of prenatal and postnatal mothers in risk factor categories or strata (i.e. presence versus absence of a characteristic) is illustrated by a 95% probability coverage indicative of a protective effect for high social supports scores on MDD (crude PPR) at SITE II. After adjustment for misclassification error, the median PPR was lower with a narrower 95%BCI indicating an attenuation effect. However, joint-adjustment for misclassification error and confounding resulted in a 95% probability coverage of the PPR that suggested a null association could not be ruled out. In this example, the proportion of mothers who had low levels social supports (i.e. 1^st^ and 2^nd^ quartiles) was 26% higher among mothers who had postnatal versus prenatal MDD assessments. Generally, although the median bias tended to increase after joint adjustment for misclassification error and confounding at both study sites (I and II), it did not do so all the time (e.g. social support at SITE II, where median bias decreased). This makes it challenging to predict the direction of bias on measures of association without valid data analysis (i.e. addressing outcome [MDD] misclassification error within categories of a confounder [prenatal versus postnatal].

Observed variations in the extent of bias on measures of association across different risk factors in our study data have been demonstrated before in a general population study. Findings from a study by Savoca [42] examining the impact of misclassification error on socio-demographic correlates of MDD using National Comorbidity Survey (NCS) data found that without adjustment for misclassified MDD diagnoses, the magnitude of the measure of association between gender and MDD (prevalence odds ratios) and marital status and MDD were underestimated by 42% and 60%, respectively. Unlike the validation approach (criterion standard based on psychiatrist diagnoses) used by Savoca, the use of the Bayesian approach in our study is a more cost efficient approach for the adjustment of misclassification error when examining epidemiological associations. That is, the Bayesian approach does not require re-administration of reference standards or use of experts to verify MDD diagnoses but rather uses existing information (as priors) to correct for and estimate impact of misclassification error on investigated associations.

Our study findings also reinforce the fact that some risk factors of MDD may be different in different source populations after adjustment for misclassification error and confounding by period of MDD assessment. Therefore, preventive interventions that are designed for specific populations may not necessary be ideal in other populations with potentially different indicators of MDD risk. As demonstrated by our results, characteristics such as adequacy of family resources may have a stronger protective association among low-income recent Hispanic immigrants (SITE II) than among US resident mothers (SITE I).

### Study limitations

One limitation of this study was that the eligibility criteria of the parent studies were such that only mothers at high risk of child maltreatment (an outcome correlated with MDD)[2] were recruited for home visitation services [43]. Therefore, because the study population may have been more homogenous than the general population of mothers, the observed magnitude of measures of association between suspected risk factors and MDD may be lower than those observed in general population studies. However, this probably had little impact on our assessment of misclassification error impact on measures of association (PPR).

Another limitation of the study is the fact that the CESD-SF uses a seven-day reference period for MDD symptoms (at baseline assessment). This probably underestimated the ‘true’ prevalence of MDD if symptoms present during 8 to 14 days prior to the interview disappeared the following week. However, because MDD symptoms (i.e. an episode of MDD) usually last for two to six months without treatment [44, 45], the impact of the short symptom reference period on risk factor associations investigated is likely small. Moreover, comparisons of sensitivity and specificity between one week and two week symptom reference period instruments have shown substantial overlap in previous studies [46].

Furthermore, the prior estimates used for the CESD-SF in our analysis are based on the assumption that the sensitivity and specificity values of the 20- and 12-item CESD instrument are comparable. Although this assumption is supported by validity equipoise between even shorter versions (2- and 5- item CESD instruments) of the 20-item CESD in previous literature [47], it is plausible that the use of CESD12 prior values may have resulted in different (but comparable) findings than those reported in this study. Ideally, while the use of prior values from a similar version of the 12-item CESD-SF is recommended, the impact of using CESD20 (versus CESD12) prior values on our findings is likely small but nonetheless cannot be ruled out.

Due to the small study sample sizes, simultaneous control for multiple potential confounding factors was not possible. This means reported misclassification adjusted PPR may be biased by potential confounders not controlled for other than period of MDD assessment. Future research should simultaneously examine the joint influence of misclassification error and confounding by other population characteristics on epidemiological associations.

Additionally, given the fact that only one test (CESD-SF) was examined in this study, the prior information suggested for sensitivity and specificity was not updated by the observed data. Moreover, the small sub-group sample sizes, especially after stratification by period of MDD assessment resulted in very uncertain posterior distribution summary estimates (i.e. wide 95% BCI for PPR estimates). Given the relatively high levels of uncertainty associated with our adjusted measures of associations (especially at SITE I), future studies or simulation studies with larger sample sizes are needed to further support recommendations for joint adjustment for misclassification error and confounding by period of MDD assessment. The use of multiple (or more than one) case-finding instrument to determine the operational definition of MDD could also be explored to reduce the level of uncertainty in observed measures of association.

It can also be argued that our study exposures or risk factors of interest (e.g. intimate partner violence) may have been misclassified. Such misclassification could be either differential (i.e. sensitivity and specificity of the violence assessment tool is different depending on a mother’s MDD status) or non-differential (i.e. sensitivity and specificity of the violence assessment tool is the same irrespective of a mother’s MDD status). Assuming non-differential misclassification of risk factors, our observed level of bias may be underestimated [48]. In contrast, the directionality and magnitude of bias under differential misclassification scenarios is not predictable. Future research (with larger sample sizes) should examine the composite effects of confounding, MDD and exposure (risk factor) misclassification on measures of association.

Despite these limitations, our study findings underscore the substantial impact differential misclassification error and confounding by period of MDD assessment can have on inferences regarding suspected MDD risk factors. Biased measures of association can result in poorly planned preventive interventions that ignore or miss etiologic risk factors related to MDD illness.

## Supporting information

S1 File WinBUGS Syntax for estimating crude and adjusted prevalence proportion ratios (PPR) summarizing the relationship between suspected risk factors and major depression disorder among mothers of young children. (PDF)

## References

[1] Gaynes BN, Gavin N, Meltzer-Brody S, Lohr KN, Swinson T, Gartlehner G, et al Perinatal depression: prevalence, screening accuracy, and screening outcomes. EvidRep Technol Assess. 2005;119:1–8. 10.1037/e439372005-001 PMC4780910 15760246

[2] Ammerman RT, Putnam FW, Bosse NR, Teeters AR, Van Ginkel JB. Maternal Depression in Home Visitation: A Systematic Review. Aggress Violent Behav. 2010;15(3):191–200. 10.1016/j.avb.2009.12.002 20401324 PMC2855144

[3] Farr SL, Hayes DK, Bitsko RH, Bansil P, Dietz PM. Depression, Diabetes, and Chronic Disease Risk Factors Among US Women of Reproductive Age. Prev Chronic Dis. 2011;8(6):A119 22005612 PMC3221561

[4] Ertel KA, Rich-Edwards JW, Koenen KC. Maternal depression in the United States: nationally representative rates and risks. J Womens Health. 2011;20(11):1609–1617. 10.1089/jwh.2010.2657 PMC3253390 21877915

[5] Goodman SH, Rouse MH, Connell AM, Broth MR, Hall CM, Heyward D. Maternal depression and child psychopathology: a meta-analytic review. Clin Child Fam Psychol Rev. 2011;14(1):1–27. 10.1007/s10567-010-0080-1 21052833

[6] Beardslee WR, Versage EM, Gladstone TR. Children of affectively ill parents: a review of the past 10 years. J Am Acad Child Adolesc Psychiatry. 1998;37(11):1134–1141. 9808924 10.1097/00004583-199811000-00012

[7] O'Hara MW, Swain AM. Rates and risk of postpartum depression-a meta-analysis. Int Rev Psychiatry. 1996;8:37–54.

[8] Lancaster CA, Gold KJ, Flynn HA, Yoo H, Marcus SM, Davis MM. Risk factors for depressive symptoms during pregnancy: a systematic review. Am J Obstet Gynecol. 2010;202(1):5–14. 10.1016/j.ajog.2009.09.007 20096252 PMC2919747

[9] Beck CT. A meta-analysis of predictors of postpartum depression. Nurs Res. 1996;45(5):297–303. 8831657 10.1097/00006199-199609000-00008

[10] Stewart DE, Robertson E, Dennis C-L, Grace SL, Wallington T. Postpartum depression: Literature review of risk factors and interventions; 2003. Available from: https://www.who.int/mental_health/prevention/…/lit_review_postpartum_depression.pdf. Cited 18 September 2018.

[11] Beck CT. Predictors of postpartum depression: an update. Nurs Res. 2001;50(5):275–285. 11570712 10.1097/00006199-200109000-00004

[12] Duggan AK, Berlin LJ, Cassidy J, Burrell L, Tandon SD. Examining Maternal Depression and Attachment Insecurity as Moderators of the Impacts of Home Visiting for At-Risk Mothers and Infants. J Consul Clin Psychol. 2009;77(4):788–799. 10.1037/a0015709 PMC2718774 19634970

[13] Tandon SD, Parillo KM, Jenkins C, Duggan AK. Formative evaluation of home visitors' role in addressing poor mental health, domestic violence, and substance abuse among low-income pregnant and parenting women. Matern Child Health J. 2005;9(3):273–283. 10.1007/s10995-005-0012-8 16240078

[14] Ammerman RT, Putnam FW, Altaye M, Chen L, Holleb LJ, Stevens J, et al Changes in depressive symptoms in first time mothers in home visitation. Child Abuse Negl. 2009;33(3):127–138. 10.1016/j.chiabu.2008.09.005 19328548 PMC2710301

[15] Stevens J, Ammerman R., Putnam F., & Van Ginkel J. Depression and trauma history in first-time mothers receiving home visitation. J Community Psychol. 2002(30):551–564.

[16] Chazan-Cohen R, Ayoub C, Pan BA, Roggman L, Raikes H, McKelvey L, et al It takes time: Impacts of Early Head Start that lead to reductions in maternal depression two years later. Infant Ment Health J. 2007;28(2):151–170. 10.1002/imhj.20127 28640556

[17] Chaffin M, Bard D, Bigfoot DS, Maher EJ. Is a Structured, Manualized, Evidence-Based Treatment Protocol Culturally Competent and Equivalently Effective Among American Indian Parents in Child Welfare? Child Maltreat. 2012;17(3):242–252. 10.1177/1077559512457239 22927674 PMC4443699

[18] Kessler RC, Bromet EJ. The epidemiology of depression across cultures. Annu Rev Public Health. 2013;34:119–138. 10.1146/annurev-publhealth-031912-114409 23514317 PMC4100461

[19] Kerr LK, Kerr LD Jr. Screening tools for depression in primary care: the effects of culture, gender, and somatic symptoms on the detection of depression. West J Med. 2001;175(5):349–352. 11694495 10.1136/ewjm.175.5.349 PMC1071624

[20] Simon GE, Goldberg DP, Von Korff M, Ustun TB. Understanding cross-national differences in depression prevalence. Psychol Med. 2002;32(4):585–594. 12102373 10.1017/s0033291702005457

[21] Pereira AT, Marques M, Soares MJ, Maia BR, Bos S, Valente J, et al Profile of depressive symptoms in women in the perinatal and outside the perinatal period: similar or not? J Affect Disord. 2014;166:71–78. 10.1016/j.jad.2014.04.008 25012412

[22] Bright JI, Baker KD, Neimeyer RA. Professional and paraprofessional group treatments for depression: a comparison of cognitive-behavioral and mutual support interventions. J Consult Clin Psychol. 1999;67(4):491–501. 10450619 10.1037//0022-006x.67.4.491

[23] Kernot J, Olds T, Lewis LK, Maher C. Test-retest reliability of the English version of the Edinburgh Postnatal Depression Scale. Arch Womens Ment Health. 2015;18(2):255–257. 10.1007/s00737-014-0461-4 25209355

[24] Chaudron LH, Szilagyi PG, Tang W, Anson E, Talbot NL, Wadkins HIM, et al Accuracy of Depression Screening Tools for Identifying Postpartum Depression Among Urban Mothers. Pediatrics. 2010;125(3):e609–e617. 10.1542/peds.2008-3261 20156899 PMC3030186

[25] Ji S, Long Q, Newport DJ, Na H, Knight B, Zach EB, et al Validity of depression rating scales during pregnancy and the postpartum period: impact of trimester and parity. J Psychiatr Res. 2011;45(2):213–219. 10.1016/j.jpsychires.2010.05.017 20542520 PMC2945623

[26] Owora AH, Carabin H. Impact of misclassification error in the estimation of maternal major depression disorder prevalence in home visitation programs. Psychiatry Res. 2018;261:80–87. 10.1016/j.psychres.2017.12.047 29289025

[27] Rothman KJ, Greenland S. Modern Epidemiology. 2nd ed. Philadelphia: Lippincott Williams and Wilkins; 1998.

[28] Brenner H, Savitz DA, Gefeller O. The effects of joint misclassification of exposure and disease on epidemiologic measures of association. J Clin Epidemiol. 1993;46(10):1195–1202. 8410104 10.1016/0895-4356(93)90119-l

[29] Caronongan P, Boller K, Vogel C, Bradley C, Barrett K, Malone L. Family and Child Outcomes Data Collection Manual for the Evidence-Based Home Visiting to Prevent Child Maltreatment Cross-Site Evaluation. Princeton (NJ): Mathematica Policy Research; 2011 5. Contract No.: GS-10F-0050L/ HHSP233200800065W. Sponsored by the Children’s Bureau, Administration for Children and Families, U.S. Department of Health and Human Services.

[30] Radloff LS. The CESD scale: a self report scale for research in the general population. Appl Psychol Meas. 1977;1:385–401.

[31] Beeghly M, Olson KL, Weinberg MK, Pierre SC, Downey N, Tronick EZ. Prevalence, stability, and socio-demographic correlates of depressive symptoms in Black mothers during the first 18 months postpartum. Matern Child Health J. 2003;7(3):157–168. 14509411 10.1023/a:1025132320321

[32] Orr ST, James SA, Blackmore Prince C. Maternal prenatal depressive symptoms and spontaneous preterm births among African-American women in Baltimore, Maryland. Am J Epidemiol. 2002;156(9):797–802. 12396996 10.1093/aje/kwf131

[33] Tandon SD, Cluxton-Keller F, Leis J, Le HN, Perry DF. A comparison of three screening tools to identify perinatal depression among low-income African American women. J Affect Disord. 2012;136(1–2):155–162. 10.1016/j.jad.2011.07.014 21864914 PMC3789596

[34] Dunst CJ, Leet HE. Measuring the adequacy of resources in households with young children. Child Care Health Dev. 1987;13(2):111–125. 3581439 10.1111/j.1365-2214.1987.tb00528.x

[35] Weiss RS. The provisions of social relationships In: Rubin Z, editor. Doing unto others. New Jersey: Prentice-Hall; 1974 p. 17–26.

[36] Straus MA, Douglas EM. A short form of the Revised Conflict Tactics Scales, and typologies for severity and mutuality. Violence Vict. 2004;19(5):507–520. 15844722 10.1891/vivi.19.5.507.63686

[37] Geweke J. Evaluating the accuracy of sampling-based approaches to calculating posterior moments In: Bernardo JM, Berger J, Dawid AP, Smith JFM, editors. Bayesian Statistics 4: Oxford University Press; 1992 p. 169–193.

[38] Heidelberger P, Welch PD. Simulation Run Length Control in the Presence of an Initial Transient. Oper Res. 1983;31:1109–1145.

[39] Gelman A, Rubin DB. Inference from Iterative Simulation Using Multiple Sequences. Stat Sci. 1992;7(4):457–472.

[40] Lunn DJ, Thomas A, Best N, Spiegelhalter DJS. WinBUGS—A Bayesian modelling framework: Concepts, structure, and extensibility. Stat Comput. 2000;10(4):325–337.

[41] Owora AH, Carabin H, Reese J, Garwe T. Summary diagnostic validity of commonly used maternal major depression disorder case finding instruments in the United States: A meta-analysis. J Affect Disord. 2016;205:335–343. 10.1016/j.jad.2016.08.014 27566453 PMC5568628

[42] Savoca E. Sociodemographic Correlates of Psychiatric Diseases: Accounting for Misclassification in Survey Diagnoses of Major Depression, Alcohol, and Drug Use Disorders. Health Serv Outcomes Res Methodol. 2004(5):175–191.

[43] Adirim T, Supplee L. Overview of the Federal Home Visiting Program. Pediatrics. 2013;132(Suppl 2):S59–S64. 24187124 10.1542/peds.2013-1021C

[44] Spijker J, de Graaf R, Bijl RV, Beekman AT, Ormel J, Nolen WA. Duration of major depressive episodes in the general population: results from The Netherlands Mental Health Survey and Incidence Study (NEMESIS). Br J Psychiatry. 2002;181:208–213. 12204924 10.1192/bjp.181.3.208

[45] Ghio L, Gotelli S, Cervetti A, Respino M, Natta W, Marcenaro M, et al Duration of untreated depression influences clinical outcomes and disability. J Affect Disord. 2015;175:224–228. 10.1016/j.jad.2015.01.014 25658495

[46] Owora AH, Carabin H, Reese J, Garwe T. Diagnostic performance of major depression disorder case-finding instruments used among mothers of young children in the United States: A systematic review. J Affect Disord. 2016;201:185–93. 10.1016/j.jad.2016.05.015 27240311 PMC5578461

[47] Shrout PE, Yager TJ. Reliability and validity of screening scales: effect of reducing scale length. J Clin Epidemiol. 1989; 42 (1): 69–78. 2913189 10.1016/0895-4356(89)90027-9

[48] Jurek AM, Greenland S, Maldonado G, Church TR. Proper interpretation of non-differential misclassification effects: expectations vs observations. Int J Epidemiol. 2005;34(3):680–687. 10.1093/ije/dyi060 15802377

