# Supplementary material for: Are we validly assessing major depression disorder risk and associated factors among mothers of young children? A cross-sectional study involving home visitation programs
Source: PLoS One. 2019 Jan 7;14(1):e0209735. doi: 10.1371/journal.pone.0209735 (PMC6322825; doi:10.1371/journal.pone.0209735)
Supplement: S1 File — (PDF) [file pone.0209735.s001.pdf]

WinBUGS Syntax for estimating crude and adjusted prevalence proportion ratios (PPR) summarizing the relationship between suspected risk factors and major depression disorder among mothers of young children

```
#Model
model
{
# prior distributions
#Below are the Se and Sp from Tandon combined periods – obtaining the alpha and beta
        mean.se <- 0.77
        se.var <- 0.12*.12
        ab.se<-(mean.se*(1-mean.se)/se.var)-1
        alpha.se<-mean.se*ab.se
        beta.se<-(1-mean.se)*ab.se

        mean.sp<-0.97
        sp.var<-0.03*0.03
        ab.sp<-(mean.sp*(1-mean.sp)/sp.var)-1
        alpha.sp<-mean.sp*ab.sp
        beta.sp<-(1-mean.sp)*ab.sp

         mean1.se <- 0.70
        se1.var <- 0.12*.12
        ab1.se<-(mean1.se*(1-mean1.se)/se1.var)-1
        alpha1.se<-mean1.se*ab1.se
        beta1.se<-(1-mean1.se)*ab1.se

        mean1.sp<-0.90
        sp1.var<-0.03*0.03
        ab1.sp<-(mean1.sp*(1-mean1.sp)/sp1.var)-1
        alpha1.sp<-mean1.sp*ab1.sp
        beta1.sp<-(1-mean1.sp)*ab1.sp

#This defines the distributions of Se and Spec
       se~dbeta(alpha.se,beta.se)
       spec~dbeta(alpha.sp,beta.sp)
       likelihood.ratio.pos <- se/(1-spec)
       likelihood.ratio.neg <- (1 - se)/spec

       se.a~dbeta(alpha1.se,beta1.se)
       spec.a~dbeta(alpha1.sp,beta1.sp)
       likelihood.ratio.pos.a <- se.a/(1-spec.a)
       likelihood.ratio.neg.a <- (1 - se.a)/spec.a

#Below are the estimates adjusted for ME
for (i in 1:2)
             {
             prev.mdd[i] ~ dbeta(1, 1)
             mdd.test[i] ~ dbin(positive.diag.prob[i],n[i])
             positive.diag.prob[i] <- prev.mdd[i] * se + (1 - prev.mdd[i]) * (1 - spec)
              }
#Below are the estimates adjusted for ME
for (i in 3:4)
             {
             prev.mdd[i] ~ dbeta(1, 1)
             mdd.test[i] ~ dbin(positive.diag.prob[i],n[i])
             positive.diag.prob[i] <- prev.mdd[i] * se.a + (1 - prev.mdd[i]) * (1 - spec.a)
```

```
            }



#Below is the observed prevalence
for (i in 1:4)
           {
            obs.prev[i] ~dbeta(1,1)
            mdd.test[i]~dbin(obs.prev[i],n[i])
            }

#Estimating the adjusted and observed PPR and bias
     adj.ppr<-(prev.mdd[1] +mdd.[3])/ (prev.mdd[2] +prev.mdd[3])
     obs.ppr<-obs.prev[1] +obs.pre/obs.prev[2]
     bias<-obs.ppr-adj.ppr

    }

DATA
list(mdd.test=c(42,22, 60, 80),n=c(150, 40, 80, 191))
```
